# High-sensitivity C-reactive protein level in stable-state bronchiectasis predicts exacerbation risk

**DOI:** 10.1186/s12890-024-02888-z

**Published:** 2024-02-13

**Authors:** Wang Chun Kwok, Kay Cheong Teo, Kui Kai Lau, James Chung-man HO

**Affiliations:** Department of Medicine, The University of Hong Kong, Queen Mary Hospital, 4/F, Professorial Block, 102 Pokfulam Road, Pokfulam, Hong Kong Special Administrative Region China

**Keywords:** Bronchiectasis, Bronchiectasis exacerbation, Phenotype, CRP, hs-CRP

## Abstract

**Background:**

Elevation of systemic inflammatory markers were found to correlate with increased disease extent, reduced lung function and higher risk of future severe exacerbations in patients with bronchiectasis. Although a significant correlation of circulating hs-CRP levels with HRCT scores and resting oxygen saturation in patients with stable-state non-cystic fibrosis (CF) bronchiectasis was suggested, there is little data on the relationship between hs-CRP and the prognosis of bronchiectasis and a lack of data on the role of hs-CRP in predicting bronchiectasis exacerbation.

**Methods:**

A prospective study was conducted on Chinese patients with non- CF bronchiectasis from 1st October to 31st December 2021. Baseline serum hs-CRP were obtained at stable-state. The follow-up period lasted for one year. Co-primary endpoints were the development of any bronchiectasis exacerbation and hospitalized bronchiectasis exacerbation.

**Results:**

Totally 123 patients were included. Higher hs-CRP was associated with increased risk to develop any bronchiectasis exacerbation, adjusted odds ratio (aOR) of 2.254 (95% CI = 1.040–4.885, *p* = 0.039), and borderline significantly increased hospitalized bronchiectasis exacerbation with aOR of 1.985 (95% CI = 0.922–4.277, *p* = 0.080).

**Conclusion:**

Baseline serum hs-CRP level at stable-state can predict risk of bronchiectasis exacerbation, which is reflecting chronic low-grade inflammation in bronchiectasis.

**Supplementary Information:**

The online version contains supplementary material available at 10.1186/s12890-024-02888-z.

## Introduction

The main pathogenesis of non-cystic fibrosis (CF) bronchiectasis involves airway inflammation, abnormal mucus clearance and bacterial colonization, resulting in progressive airway destruction and distortion [1]. The classical type of airway inflammation is neutrophilic, with abundance of neutrophils in sputum, bronchoalveolar lavage fluid and bronchial biopsy from patients with non-CF bronchiectasis, even in clinically stable-state [2]. The recruitment and trafficking of neutrophils to bronchiectatic airways are mediated via various pro-inflammatory cytokines like interleukin-1β (IL-1β), IL-8, tumor necrosis factor (TNF)-α and leukotriene (LT) B4 [3]. In previous studies, airway neutrophilic inflammation as indicated by sputum neutrophil count was inversely correlated with lung function (forced expiratory volume in 1 s [FEV_1_]) and directly with duration of disease and severity (Bronchiectasis Severity Index [BSI]) in stable non-CF bronchiectasis [4, 5]. It was also demonstrated that sputum elastase, released from airway neutrophils, significantly correlated with 24-hour sputum volume, number of bronchiectatic lobes, percent predicted FEV_1_, and sputum leukocyte count in stable-state bronchiectasis [5]. Patients with non-CF bronchiectasis harbouring *Pseudomonas aeruginosa* showed greater sputum neutrophilia and volume, with lower FEV_1_ and FEV_1_/forced vital capacity (FVC) ratio in previous studies from our group and others [4, 5].

Elevation of systemic inflammatory markers including C-reactive protein (CRP) and total white cell count, has been found to correlate with the extent of the disease and poor lung function in bronchiectasis [6]. The CRP value was associated with a greater risk of future severe exacerbations but not with mild or moderate exacerbations in patients with steady-state bronchiectasis from the Spanish Registry of Bronchiectasis (RIBRON) [7].

High-sensitivity CRP (hs-CRP) is a simple blood test that is readily available in many healthcare facilities. It is also repeatable and serial measurements are feasible. Hs-CRP also has the advantage over CRP that it can detect low-grade inflammation, which may be present in chronic inflammatory conditions such as bronchiectasis. As an inflammatory marker, hs-CRP has been extensively studied in cardiovascular diseases [8–12]. There is little data on the level of hs-CRP and its relationship with the prognosis in bronchiectasis. One study suggested that circulating hs-CRP levels were significantly correlated with HRCT scores and resting oxygen saturation in patients with stable non-CF bronchiectasis [13]. However, there is a lack of data on the role of hs-CRP in predicting bronchiectasis exacerbation, which is associated with negative impact on morbidity, mortality, quality of life, and health care costs [14–22]. In this study, we aim to assess the association between hs-CRP level and the risks of bronchiectasis exacerbation.

## Materials and methods

This was a prospective clinical study in patients with bronchiectasis conducted in Queen Mary Hospital (QMH), Hong Kong. Patients with non-CF bronchiectasis followed up in the adult Respiratory Specialty Clinic at QMH were recruited from 1st October to 31st December 2021. Written informed consent was obtained from each eligible subject. A total of 166 patients with bronchiectasis were recruited, among which 123 had serum hs-CRP results available at stable-state.

QMH is one of the major regional hospitals in Hong Kong and a University-affiliated tertiary respiratory referral centre, with a designated bronchiectasis clinic for managing patients with non-CF bronchiectasis of different disease severity. The investigators reviewed clinic records and radiographic findings to validate the diagnosis of bronchiectasis. Patients’ records were accessed through the Electronic Patient Record (ePR) system of the Hong Kong Hospital Authority, which comprised both outpatient and in-patient episodes. The information available included demographics, clinical notes, investigation results and treatments.

The inclusion criteria included Chinese patients with non-CF bronchiectasis age at or above 18 years old. The diagnosis of bronchiectasis was confirmed by high resolution computed tomography findings of bronchiectasis that were verified by radiologists. Exclusion criteria included cystic fibrosis (which is extremely rare in Chinese population with only 46 reported cases out of 1.4 billion population) [23], traction bronchiectasis from interstitial lung disease and individuals lost to follow-up. Demographic data (age, gender, smoking status), clinical data / investigations (etiology of bronchiectasis, comorbidities, treatment records, spirometry results, sputum culture results) were collected. Baseline serum hs-CRP level was taken at clinically stable-state, which was defined as at least 3 months away from bronchiectasis exacerbation. Patients were recruited only if their last bronchiectasis exacerbation requiring antibiotics treatment was at least 3 months before the study recruitment.

E-FACED score which comprised E (at least one severe exacerbation in previous one year), F (forced expiratory volume in 1 s [FEV_1_]), A (age), C (chronic colonization by Pseudomonas aeruginosa), E (radiological extent [number of pulmonary lobes affected]), and D (dyspnea) was calculated for the patients included [24].

The primary outcome was any bronchiectasis exacerbation within the one-year follow-up period. Bronchiectasis exacerbation was defined as (1) a deterioration in three or more of the key symptoms (including cough, sputum volume and/or consistency, sputum purulence, dyspnea and/or exercise tolerance, fatigue and/or malaise, and hemoptysis) for at least 48 h and (2) clinician’s assessment that a change in treatment was required [25]. The secondary outcome was hospitalized bronchiectasis exacerbation within the study period, which is defined as bronchiectasis exacerbation that requited in-patient stay for at least 24 h, as well as correlating hs-CRP level with severity of bronchiectasis and inflammatory markers by neutrophil-to-lymphocyte ratio (NLR). Patients who had bronchiectasis exacerbations requiring in-patient care were identified from the ePR. The study was approved by the Institutional Review Board of The University of Hong Kong and Hospital Authority Hong Kong West Cluster (approval number UW19-624).

### Statistical analysis

The demographic and clinical data were described in actual frequency or mean ± SD. Baseline demographic and clinical data were compared between the two groups (with or without bronchiectasis exacerbation) with independent t-tests. Logistic regression was used to estimate the risk of bronchiectasis exacerbation with different hs-CRP levels at stable-state in the 1-year follow-up period. E-FACED score [24], gender and smoking status were adjusted as potential confounders. Associations between hs-CRP level and the number of bronchiectasis exacerbation were assessed with the use of linear regression, with adjustment for potential confounders.

The relationship between hs-CRP level, severity of bronchiectasis as measured by FEV_1_, mMRC dyspnea scale, number of lobes involved, FACED score, E-FACED score and inflammatory markers by NLR were assessed using the Pearson’s correlation coefficient metrics. Statistical significance was determined at the level of *p* = 0.05. All statistical analyses were performed using the 28th version of SPSS statistical package.

Based on the previous study on the role of CRP and bronchiectasis exacerbation risk [7], a sample size of 94 was needed for a 90% power to detect a difference between matched pairs using a two-sided type I error of 0.05.

## Results

### Baseline and clinical characteristics

A total of 123 Chinese patients with non-CF bronchiectasis were included. The mean age was 68.5 ± 11.2 years. There were more females (65%) and never-smokers (84.6%). *Pseudomonas aeruginosa* was the commonest colonizer in sputum (35.8%). The mean FEV_1_ was 1.71 ± 0.64 L (87.3 ± 23.2%). Multilobar involvement, defined as those with disease in more than 3 lobes, were seen in 48 (39.0%) of the patients. The median E-FACED score was 2 [Interquartile range (IQR) = 0.5–3]. The mean hs-CRP level at stable-state was 0.37 ± 0.64 mg/dL. There were 26 and 10 patients who developed any bronchiectasis exacerbation and hospitalized bronchiectasis exacerbation during the follow-up period. The results are summarized in Table 1.

**Table 1:** Baseline demographic and clinical characteristics

	Whole cohort (*n* = 123)	No exacerbation on follow-up (*n* = 97)	Exacerbation on follow-up (*n* = 26)	P-values
Age (years), mean ± SD	68.5 ± 11.2	68.7 ± 10.4	67.9 ± 14.0	0.395
Male	43 (35.0%)	30 (30.9%)	13 (50.0%)	0.070
Smoking status				0.833
Current smoker	4 (3.3%)	3 (3.1%)	1 (3.8%)	
Ex-smoker	15 (12.2%)	11 (11.3%)	4 (15.4%)	
Non-smoker	104 (84.6%)	83 (85.6%)	21 (80.8%)	
FEV_1_ (L), mean ± SD	1.71 ± 0.64	1.69 ± 0.60	1.79 ± 0.76	0.303
FEV_1_ (% predicted) mean ± SD	87.3 ± 23.2	90.0 ± 22.8	78.0 ± 22.9	0.019*
FEV_1_/FVC ratio (%), mean ± SD	68.8 ± 11.9	69.4 ± 11.4	66.6 ± 13.6	0.190
mMRC dyspnea scale				0.308
0	27 (22.0%)	24 (24.7%)	3 (11.5%)	
1	45 (36.6%)	35 (36.1%)	10 (38.5%)	
2	39 (31.7%)	31 (32.0%)	8 (30.8%)	
3	10 (8.1%)	6 (6.2%)	4 (15.4%)	
4	2 (1.6%)	1 (1.0%)	1 (3.8%)	
Extent of involvement ≥ 3 lobes	48 (39.0%)	35 (36.1%)	13 (50.0%)	0.196
*Pseudomonas aeruginosa* colonization	44 (35.8%)	29 (29.9%)	15 (57.7%)	0.009*
hs-CRP (mg/dL), mean ± SD	0.37 ± 0.64	0.28 ± 0.44	0.71 ± 1.04	0.026*
E-FACED score, median [IQR]	2 [0.5–3]	2 [0–3]	2 [1–4]	0.158

SD = standard deviation; mL = milliliter; * = statistically significant; FEV_1_ = forced expiratory volume in one second; FVC = forced vital capacity

**Fig. 1:**
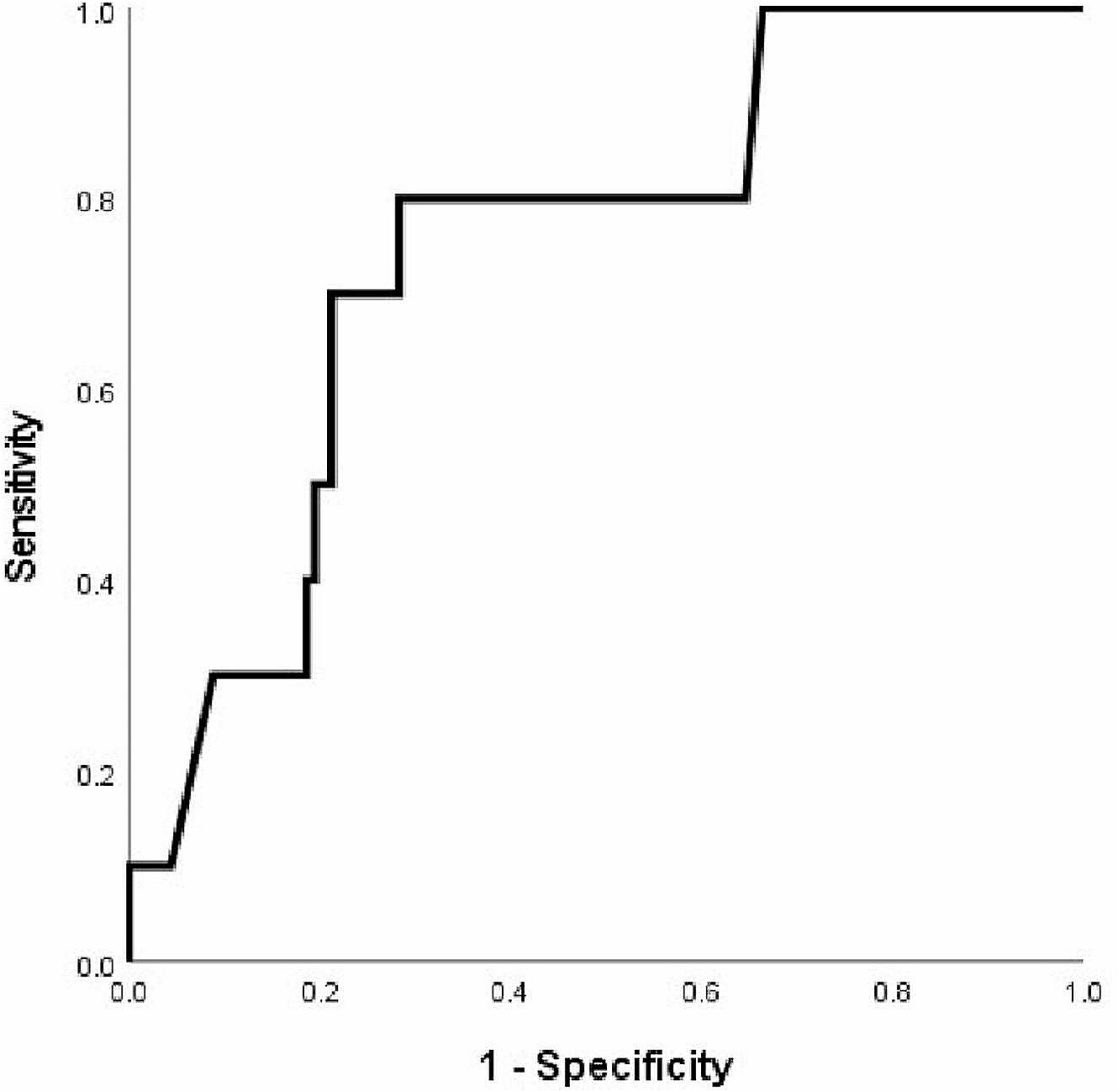
Receiver Operating Curve (ROC) for hs-CRP-S and risks of hospitalized bronchiectasis exacerbation in 1 year follow-up

By receiver operator curve (ROC) analysis, hs-CRP level of 0.3 mg/dL had sensitivity of 80% and specificity of 71.7%, with the area under the curve of 0.747 (95% CI = 0.603–0.891, *p* = 0.010) (Fig. 1).

### Risk of any bronchiectasis exacerbation

The mean number of bronchiectasis exacerbation was 0.27 ± 0.57 for the whole cohort. Univariate regression analysis showed that higher hs-CRP was associated with significantly higher risk of any bronchiectasis exacerbation with odds ratio (OR) of 2.597 (95% confidence interval [CI] = 1.188–5.676, *p* = 0.017). The result remained significant after adjusting for E-FACED score, gender, and smoking status, with adjusted odds ratio (aOR) 2.365 (95% CI = 1.042–5.364, *p* = 0.039). Using a defined cut-off, hs-CRP > 0.35 mg/dL, which was the cut-off level in the QMH immunology laboratory to define normal hs-CRP value, had significantly higher risk to develop any bronchiectasis exacerbation with OR of 4.222 (95% CI = 1.700–4.885, *p* = 0.002) and aOR of 3.915 (95% CI = 1.423–10.771, *p* = 0.008). hs-CRP level was positively correlated with the number of bronchiectasis exacerbation with *r* = 0.334, *p* < 0.001. Patients with hs-CRP > 0.35 had significantly more bronchiectasis exacerbation than those with hs-CRP ≤ 0.35, with mean number of exacerbations being 0.54 ± 0.78 in the high hs-CRP group and 0.16 ± 0.43 in the low hs-CRP group, *p* = 0.001 in both univariate and multivariate analysis. The results are summarized in Table 2.

### Risk of hospitalized bronchiectasis exacerbation

The mean number of bronchiectasis exacerbation was 0.11 ± 0.40 for the whole cohort. Univariate regression analysis showed that higher hs-CRP was associated with significantly higher risk to develop hospitalized bronchiectasis exacerbation with OR of 2.280 (95% CI = 1.107–4.695, *p* = 0.025). The result was not significant after adjusting for E-FACED score, gender and smoking status, with aOR of 2.031 (95% CI = 0.944–4.367, *p* = 0.070). Using a defined cut-off, hs-CRP > 0.35 mg/dL, the cut-off level in the QMH immunology laboratory to define normal hs-CRP value, had significantly higher risk to develop hospitalized bronchiectasis exacerbation with OR of 7.083 (95% CI = 1.715–29.256, *p* = 0.007) and aOR of 4.663 (95% CI = 1.047–20.772, *p* = 0.043). hs-CRP level was positively correlated with the number of hospitalized bronchiectasis exacerbation with *r* = 0.231, *p* = 0.010. Patients with hs-CRP > 0.35 had significantly more bronchiectasis exacerbation than those with hs-CRP ≤ 0.35, with mean number of exacerbations being 0.29 ± 0.67 in the high hs-CRP group and 0.03 ± 0.18 in the low hs-CRP group, *p* = 0.001 in univariate analysis and 0.015 in multivariate analysis. The results are summarized in Table 2.

**Table 2:** Risks of bronchiectasis exacerbation based on baseline hs-CRP level at stable-state

	Univariate logistic regression			Multivariate logistic regression#		
	OR	95% CI	p-value	OR	95% CI	p-value
**Any bronchiectasis exacerbation**						
Hs-CRP as continuous variable	2.597	1.188–5.676	0.017*	2.365	1.042–5.364	0.039*
Hs-CRP above 0.35 mg/dL	4.222	1.700–4.885	0.002*	3.915	1.423–10.771	0.008*
**Hospitalized bronchiectasis exacerbation**						
Hs-CRP as continuous variable	2.280	1.107–4.695	0.025*	2.031	0.944–4.367	0.070
Hs-CRP above 0.35 mg/dL	7.083	1.715–29.256	0.007*	4.663	1.047–20.772	0.043*

#: Adjusted for smoking status, gender and E-FACED score

*: *p* < 0.05

### Correlating hs-CRP with severity of bronchiectasis and inflammatory markers

Hs-CRP level had significant positive correlation with number of lobes involved (*r* = 0.293, *p* = 0.001), FACED score (*r* = 0.236, *p* = 0.016), E-FACED score (*r* = 0.214, *p* = 0.028) and NLR (*r* = 0.659, *p* < 0.001). Hs-CRP did not have significant correlation with mMRC dysnpoea scale (*r* = 0.157, *p* = 0.082), FEV_1_ (Litre) (*r* = -0.055, *p* = 0.587) and FEV_1_ (% predicted) (*r* = -0.145, *p* = 0.0149).

### Sensitivity analysis

Sensitivity analysis was performed with the factors adjusted in multivariate analysis gender, smoking status and individual components in E-FACED score (age, FEV_1_ [% predicted], mMRC dyspnea scale, extent of bronchiectasis and number of severe exacerbations in past year as continuous variables; chronic colonization by *Pseudomonas aeruginosa* as categorical variable).

The sensitivity analysis showed largely consistent results and was summarized in supplementary Table S2.

## Discussion

In this single center prospective study, elevated serum hs-CRP level at stable-state was found to be associated with increased risks of bronchiectasis exacerbation. The level of baseline hs-CRP may possibly reflect the degree of low-grade chronic inflammation in bronchiectasis which eventually be translated into exacerbation risk.

Bronchiectasis exacerbation is one of the key factors in the assessment of severity and prognosis of bronchiectasis. It is also associated with adverse outcomes. The most readily available scoring system in bronchiectasis incorporates the background demographics, lung function impairment, sputum microbiology, extent of bronchiectasis, symptom burden as well as bronchiectasis exacerbation history in determining the prognosis [14, 24, 26]. While these scores represent the severity of bronchiectasis which is associated with prognosis, they did not incorporate the degree of airway inflammation into consideration. To have a more dynamic assessment of the disease status, incorporation of inflammatory markers that could vary according to disease activity into these scoring systems may be helpful to provide a more comprehensive assessment of disease status. For airway diseases, measuring inflammatory markers from respiratory samples such as sputum inflammatory markers would be best option [27]. While the degree of airway inflammation is difficult to detect and costly, using surrogate markers or other systemic markers such as hs-CRP would be an important and more feasible alternative. Measuring sputum inflammatory markers and cytokines are tedious and costly, requiring dedicated storage, special reagents, and dilution protocol that is labour-intensive [27, 28]. Measuring blood inflammatory markers would be a cheaper and more readily available option. Previous studies suggested the association between blood inflammatory markers at stable-state and the severity as well as exacerbation risks in bronchiectasis [7, 24, 26]. However, blood neutrophil count and CRP level both had their limitations. Absolute neutrophil count could be affected by various acute conditions including non-respiratory tract infections and systemic stress. Given the large degree of variation, it would be difficult to identify a reliable cut-off value of neutrophil count with a single measurement. Blood neutrophil percentage may resolve some of the above limitations. Conventional CRP is more specific than blood absolute neutrophil count, but the cut-off value of normal CRP level may not be sensitive enough to capture lower-grade inflammation. Hs-CRP, as determined using an assay designed to measure and distinguish very low levels of CRP close to normality, has the advantage over CRP in detecting low-grade systemic inflammation with higher precision. Hs-CRP has been shown to be associated with HRCT scores and resting oxygenation saturation in a Taiwanese study but the association with bronchiectasis exacerbation risks was not studied [13].

The findings from our study suggest the possible role of hs-CRP as a marker of low-grade systemic inflammation in stable-state that predicts subsequent bronchiectasis exacerbation. The hs-CRP was reported to be 0.10 ± 0.02 mg/dL in healthy subject [29], 0.107 mg/dL in inflammatory bowel disease (IBD) in remission and 0.899 in active IBD [30]. The hs-CRP at stable-state bronchiectasis as reported in our study was numerically higher than that in healthy subjects and IBD in remission, but lower than in patients with active IBD. Our postulation is that among patients with elevated hs-CRP level at stable-state, they have a higher degree of chronic low-grade systemic and airway inflammation. The existence of chronic low-grade systemic and airway inflammation can possibly predict the occurrence of subsequent bronchiectasis exacerbation in the 1-year follow-up period as shown in our study. Our notion is also consistent with previous studies on immunomodulatory agents and their role in preventing bronchiectasis exacerbation [31–33]. Macrolide works by modulating neutrophil inflammation in bronchiectasis and was shown to be effective in preventing bronchiectasis exacerbation. This is consistent with our finding that the degree of chronic inflammation at stable-state in bronchiectasis as measured by hs-CRP is associated with the exacerbation risks. Hs-CRP was also shown to be able to predict bronchiectasis exacerbation when it is measured as a continuous variable and as categorical variable with a cut-off value. The optimal cut-off of hs-CRP in longer term bronchiectasis exacerbation risk and mortality are worth exploring in future longer follow-up study especially in a larger population. It is also worthwhile to consider incorporating hs-CRP into various scoring systems to increase the predicting power of adverse outcomes in bronchiectasis, given its value in detecting low-grade chronic inflammation.

There are a few limitations in our study. First, this study involved only a single centre. However, being a tertiary medical centre, the respiratory unit of QMH received referrals from all other health care facilities across the territory. Patients diagnosed with bronchiectasis were managed in a designated bronchiectasis clinic in our centre. Second, all patients in this study only had a single measurement of hs-CRP at stable-state. Variability of within-person hs-CRR value has been reported [34]. Another prospective study with hs-CRP measurement at multiple time points may be conducted in future to assess the variability of hs-CRP, which may suggest varying degree of inflammation, as a better predictor of bronchiectasis exacerbation risks. Using multiple hs-CRP measurements, it may allow clinicians to categorize the patients into various subgroups. A longer duration prospective study with larger sample size is also worth considering. While hs-CRP has been assessed in this study for its role in predicting bronchiectasis exacerbation, other inflammatory markers such as plasma IL-1β, IL-8, TNF-α, and LTB4 were not examined as they were not routinely available in our centre. It is worthwhile to assess the correlation of hs-CRP with these markers, as well as whether these markers can also predict bronchiectasis exacerbation risks. Nonetheless, with the availability of plasma hs-CRP in most health care facilities, our findings can be more readily applicable across different regions.

## Conclusion

Baseline serum hs-CRP level at stable-state can predict bronchiectasis exacerbation in the subsequent year. This is in support of the role of chronic low-grade inflammation in bronchiectasis, which is reflected by hs-CRP level, in association with bronchiectasis exacerbation risk.

### Electronic supplementary material

Below is the link to the electronic supplementary material.

Supplementary Table S1 Baseline demographic and clinical characteristics; Supplementary Table S2 Risks of bronchiectasis exacerbation based on baseline hs-CRP level at stable-state, adjusted for gender, smoking status and individual components of E-FACED score

## Data Availability

All data generated or analysed during this study are included in this published article.

## Abbreviations

BS: Bronchiectasis Severity Index
CRP: C-reactive protein
CF: Cystic fibrosis
ePR: Electronic Patient Record
FEV_1_: Forced expiratory volume in 1 s
FVC: Forced vital capacity
HRCT: High-resolution computed tomography
IL-1β: Interleukin-1β
LT: Leukotriene
OR: Odds ratio
TNF-α: Tumor necrosis factor α
