# Supplementary material for: High-sensitivity C-reactive protein level in stable-state bronchiectasis predicts exacerbation risk
Source: BMC Pulm Med. 2024 Feb 13;24:80. doi: 10.1186/s12890-024-02888-z (PMC10863114; doi:10.1186/s12890-024-02888-z)
Supplement: Supplementary file 1 — Supplementary Table S1 Baseline demographic and clinical characteristics; Supplementary Table S2 Risks of bronchiectasis exacerbation based on baseline hs-CRP level at stable-state, adjusted for gender, smoking status and individual components of E-FACED score [file 12890_2024_2888_MOESM1_ESM.docx]

**Supplementary Table S1 Baseline demographic and clinical characteristics**

|  | Whole cohort  (n = 123) | Low hs-CRP ≤ 0.35  (n = 88) | High hs-CRP >0.35  (n = 35) | P-values |
| --- | --- | --- | --- | --- |
| Age (years), mean ± SD | 68.5 ± 11.2 | 67.9 ± 10.1 | 70.1 ± 13.7 | 0.391 |
| Male | 43 (35.0%) | 32 (36.4%) | 11 (31.4%) | 0.605 |
| Smoking status |  |  |  | 0.274 |
| Current smoker | 4 (3.3%) | 4 (4.5%) | 0 (0%) |  |
| Ex-smoker | 15 (12.2%) | 9 (10.2%) | 6 (17.1%) |  |
| Non-smoker | 104 (84.6%) | 75 (85.2%) | 29 (82.9%) |  |
| FEV_1_ (L), mean ± SD | 1.71 ± 0.64 | 1.76 ± 0.60 | 1.61 ± 0.72 | 0.292 |
| FEV_1_ (% predicted) mean ± SD | 87.3 ± 23.2 | 90.1 ± 21.4 | 81.5 ± 26.0 | 0.108 |
| FEV_1_/FVC ratio (%), mean ± SD | 68.8 ± 11.9 | 698.3± 11.3 | 69.8 ± 13.1 | 0.600 |
| mMRC dyspnea scale |  |  |  | 0.083 |
| 0 | 27 (22.0%) | 23 (26.1%) | 4 (11.4%) |  |
| 1 | 45 (36.6%) | 32 (36.4%) | 13 (37.1%) |  |
| 2 | 39 (31.7%) | 27 (30.7%) | 12 (34.3%) |  |
| 3 | 10 (8.1%) | 6 (6.8%) | 4 (11.4%) |  |
| 4 | 2 (1.6%) | 0 (0%) | 2 (5.7%) |  |
| Extent of involvement ≥ 3 lobes | 48 (39.0%) | 27 (30.7%) | 21 (60.0%) | 0.003* |
| *Pseudomonas aeruginosa* colonization | 44 (35.8%) | 25 (28.4%) | 19 (54.3%) | 0.007* |
| E-FACED score, median [IQR] | 2 [0.5 – 3] | 2 [0 - 3] | 3 [2-4] | 0.011* |

SD = standard deviation; mL = milliliter; * = statistically significant; FEV_1_ = forced expiratory volume in one second; FVC = forced vital capacity

**Supplementary Table S2 Risks of bronchiectasis exacerbation based on baseline hs-CRP level at stable-state, adjusted for gender, smoking status and individual components of E-FACED score**

|  | **Multivariate logistic regression** | | | | |
| --- | --- | --- | --- | --- | --- |
|  | **OR** | | **95% CI** | | **p-value** |
| **Any bronchiectasis exacerbation** |  | | | | |
| Hs-CRP as continuous variable | 2.646 | 1.012 – 6.921 | | 0.047* | |
| Hs-CRP above 0.35 mg/dL | 4.298 | 1.317 – 14.031 | | 0.016* | |
| **Hospitalized bronchiectasis exacerbation** |  | | | | |
| Hs-CRP as continuous variable | 6.506 | 0.659 – 64.194 | | 0.109 | |
| Hs-CRP above 0.35 mg/dL | 7.980 | 0.8.37 – 76.044 | | 0.071 | |

*: p < 0.05
